# Scientific Illiteracy and the Partisan Takeover of Biology

**DOI:** 10.1371/journal.pbio.0040167

**Published:** 2006-04-18

**Authors:** Liza Gross

## Abstract

Americans have long worried about conflicts between scientific and religious beliefs. For the most part, this ambivalence has not threatened the practice and teaching of biology--until recently.

Americans have long been ambivalent about science. Conflicting attitudes toward science are not uncommon among industrialized countries—Canadians, Europeans, and Japanese, for example, also appreciate the benefits of science but worry about potential impacts on society. What sets Americans apart is that their reservations center primarily around religion. And now, as the United States struggles to maintain its undisputed position as world leader in science and technology, religious ideology has spilled over into the public sphere to a degree unmatched in other industrialized societies. Religious groups are turning scientific matters like stem cells and evolution into political issues.

Though some see the growing influence of ideology over scientific issues as a threat to America's standing as global science leader, a leading analyst of public attitudes toward science sees it as an opportunity for increasing scientific literacy. “Even though the scientific community can feel besieged by this anti-science sentiment,” says Jon D. Miller, who directs the Center for Biomedical Communications at Northwestern University Medical School, “most people really haven't made up their mind about this issue and, in fact, really haven't even thought about it.” Rather than fretting about the cultural divide—or worse, doing nothing—Miller urges scientists to do their part to bridge the gap.

Since 1979, the proportion of scientifically literate adults has doubled—to a paltry 17%.

Miller has devoted his 30-year career to studying public understanding of science and technology and its implications for a healthy democracy. To possess what Miller calls civic scientific literacy, one must have the capacity to make sense of competing arguments in a scientific debate. Over the year leading up to the 2004 US election, Miller polled a national panel of adults to track their grasp of the ongoing debate about stem-cell research. A year before the election, over a third of adult respondents had never heard the term, even though the issue had dominated the headlines. By the eve of the election, only a few more respondents said they had heard about stem cells. How could so many people manage to remain oblivious to one of the most contentious issues of the election?

Most people don't have a cognitive framework for understanding stem cells, Miller explains. “Science happens so fast now that most adults couldn't possibly have learned about stem cells when they were in school.” And without this underlying schema, most people aren't going to pay attention to stem cells or any other unfamiliar scientific term. “People tune out things that they think are scientific or complicated,” he says. “If you are science averse and think you couldn't possibly know any science, the minute you hear ‘cell,’ ‘stem cell,’ ‘nanotechnology,’ ‘atomic,’ ‘nuclear,’ you turn the off switch.” [Fig pbio-0040167-g001]

**Figure pbio-0040167-g001:**
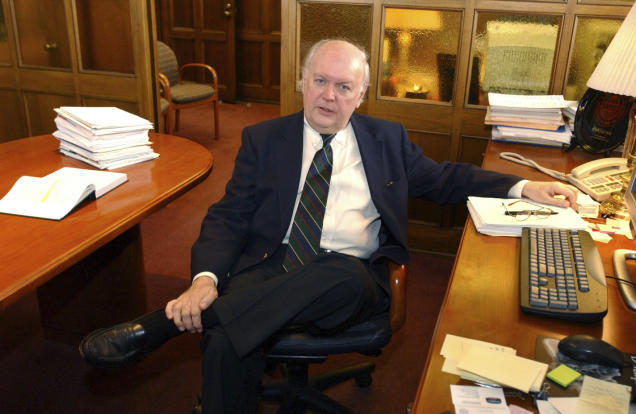
Jon D. Miller hopes that scientists will become politically engaged and oppose candidates who attack scientific research (Photo: Steve Kagan)

As time went on, more people *said* they had a good understanding of stem cells—21% in 2004, up from 9% in 2003—but only 9% of respondents could define the term when asked, compared with 8% in 2003. And, surprisingly, the number of voters with strong opinions dropped significantly. A year before the election, 17% were opposed—“likely reflecting the influence of religious groups”—and 15% were in favor. As discussions raised distinctions between adult and embryonic stem cells and between morality and scientific benefits, most people realized the issue was more complex than they had originally thought. “At the end of the election, only 2% were strongly opposed and only 2% were strongly in favor,” Miller says. “It shows that a little bit of scientific literacy won't solve the problem when you have a debate.”

For two decades, Miller served as principal investigator of the public attitudes section of the *Science and Engineering Indicators*, a biennial national report on public understanding of science and technology published by the National Science Board (which serves as the governing board of the National Science Foundation and as the national science policy advisor to the US Congress and president). Since 1979, he says, the proportion of scientifically literate adults has doubled—to a paltry 17%. The rest are not savvy enough to understand the science section of *The New York Times* or other science media pitched at a similar level. As disgracefully low as the rate of adult scientific literacy in the United States may be, Miller found even lower rates in Canada, Europe, and Japan—a result he attributes primarily to lower university enrollments.

Scientific literacy doesn't call for a deep understanding of Maxwell's equations or Hardy–Weinberg equilibrium, but it does require a general understanding of basic scientific concepts and the nature of scientific inquiry. The first national survey of American scientific literacy was serendipitously completed two months prior to the launch of Sputnik 1 in 1957, providing a baseline measure of public attitudes toward science just before the space race. People were asked about strontium 90 (a radioactive by-product of nuclear weapons tests) and the Salk vaccine—terms that dominated the newspapers at the time. But when Miller tried to replicate the survey 20 years later, it was clear that questions based on topical headlines couldn't produce accurate measures of scientific literacy over time. “You need to find more enduring measures,” he says. So he developed questions focusing on atoms, molecules, and other basic concepts, and discovered that “a lot of people have no clue about these things.”

“When I first started asking about DNA,” he says, “I used an open-ended question that asks, ‘If you saw the term *DNA* in a newspaper, would you have a clear understanding of what that means, a general sense of what it means, or not much idea?’” If respondents said they had a clear understanding, they would be asked to define DNA in their own words. “I got things like the ‘Dow Jones News Association,’” Miller says, laughing. “If you don't know what DNA is, you can't follow the stem-cell debate.”

## Measuring Attitudes toward Evolution

When it comes to monitoring and interpreting public attitudes toward science and technology, leaders in national science organizations like the National Science Foundation, the American Association for the Advancement of Science (AAAS), and the National Academies of Science, Engineering, and Medicine have long turned to Miller, an avuncular political scientist who has also advised the European Commission as well as government agencies in China, Japan, and a host of other countries. With the recent spate of high-profile lawsuits aimed at getting evolution out of public classrooms and ongoing opposition to stem-cell research, his services have been in especially high demand. Miller has long been worried about a burgeoning anti-science movement. The current climate is even more troubling, he says, with the emergence of organized attacks on science against the backdrop of the new culture wars.

To measure public acceptance of the concept of evolution, Miller has been asking adults if “human beings, as we know them, developed from earlier species of animals” since 1985. He and his colleagues purposefully avoid using the now politically charged word “evolution” in order to determine whether people accept the basics of evolutionary theory. Over the past 20 years, the proportion of Americans who reject this concept has declined (from 48% to 39%), as has the proportion who accept it (45% to 40%). Confusion, on the other hand, has increased considerably, with those expressing uncertainty increasing from 7% in 1985 to 21% in 2005.

One-third of Americans think evolution is “definitely false.”

Because simple true–false questions exaggerate the strength of both positions, Miller also asked more nuanced questions in 1993 and 2003. Again, the proportion of adults holding tentative or uncertain positions increased, but the percentage holding strong positions remained steady over the past 10 years. One-third of Americans think evolution is “definitely false”; over half lean one way or another or aren't sure. Only 14% expressed unequivocal support for evolution—a result Miller calls “shocking.”

In a graph showing acceptance (represented by blue bars) and rejection (represented by red bars) of evolution in nine European countries and the United States from a recent survey Miller conducted, red bars for the United States overshadow the blue bars for every other country. And in a 2005 survey measuring the proportion of adults who accept evolution in 34 European countries and Japan, the United States ranked 33rd, just above Turkey. No other country has so many people who are absolutely committed to rejecting the concept of evolution, Miller says. “We are truly out on a limb by ourselves.” [Fig pbio-0040167-g002]

**Figure pbio-0040167-g002:**
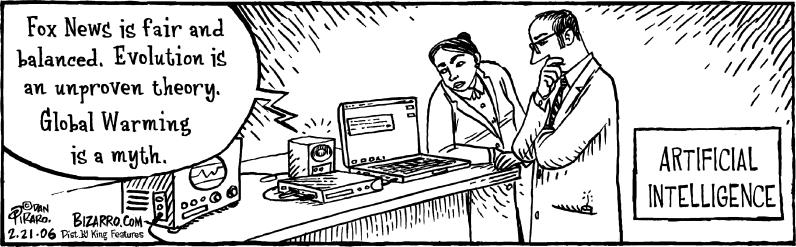
(Image: Copyright Dan Piraro, King Features Syndicate)

It's not that Americans are rejecting science per se, Miller maintains, but longstanding conflicts between personal religious beliefs and selected life-science issues has been exploited to an unprecedented degree by the right-wing fundamentalist faction of the Republican Party. In the 1990s, the state Republican platforms in Alaska, Iowa, Kansas, Oklahoma, Oregon, Missouri, and Texas all included demands for teaching creation science. Such platforms wouldn't pass muster in the election, Miller says, but in the activist-dominated primaries, they drive out moderate Republicans, making evolution a political litmus test. Come November, the Republican candidate represents a fundamentalist agenda without making it an explicit part of the campaign. Last year, Miller points out, former Senator John Danforth, a moderate Missouri Republican, wrote in a *New York Times* opinion piece that for the first time in American history a political party has become an arm of a religious organization. The United States is the only country in the world where a political party has taken a position on evolution.

To gauge the extent of fundamentalism's reach into American life, Miller evaluated adults' responses to three statements: the Bible is the actual word of God and is to be taken literally; there is a personal God who hears the prayers of individual men and women; and human beings were created by God as whole persons and did not evolve from earlier forms of life. In 2005, 43% of American adults agreed with all three statements.

“The age of nonpartisan science is gone.”

The era of nonpartisan science is gone, says Miller, who urges scientists and science educators to learn the rules of this new game and get behind moderate Republicans as well as Democrats to protect the practice and teaching of sound science. Given the partisan attack on evolution and stem-cell research, he thinks scientists need to learn more about how the political process works. They need to be willing to run for the school board, write $500 or even $5,000 checks to support moderate candidates, and defeat Christian right-wing candidates. “Scientists need to become involved in partisan politics and to oppose candidates who reject evolution or attack scientific research,” he says. “It takes time, money, and paying attention to the issues.”

## Looking Ahead

Clearly, increasing scientific literacy is a long-term challenge. The US pre-collegiate science and math education system is broken. US high-school student performance ranks behind every European and Asian country, according to the 2003 Trends in International Math and Science Study conducted by the National Center for Education Statistics. Given that over half of high-school graduates don't go on to get college degrees, that's something to be concerned about. But Miller takes heart from the fact that, unlike any other country in the world, the United States requires the 47% of kids who do go to college to take a year of science—a distinction that may help the United States recover its flagging scientific standing. College professors would do well to remember that today's undergraduates are apt to be functioning 40 to 50 years from now, he says. “It's the last chance to teach people who are going to become important leaders in the community, and we should take this opportunity seriously.”

For all the sobering statistics and challenges ahead, Miller remains undaunted. While it's unlikely that hard-core anti-evolutionists and stem-cell opponents will change their minds anytime soon, he says, “we've got a lot of ambiguity in the middle. The game is still in play. We ought not to say, ‘Gee, Americans are stupid,’ but, ‘There are a lot of Americans who would be willing to listen to us if we were to go out and make good arguments.’”

And as Miller's research shows, when you get away from the religiously charged issues, there is an even greater opportunity to increase scientific literacy. In a new study that investigates models of adult informal learning, now under review at a leading journal, he shows that public understanding of antibiotics increased substantially over a 17-year period. In 1988, just 26% of adults in a national survey knew that antibiotics do not kill both viruses and bacteria. By 2005, 54% knew that antibiotics kill only bacteria—an increase that Miller attributes to informal learning through a variety of sources.

Miller sees opportunities for learning everywhere and may be one of the few people in the scientific community to see an upside to Big Pharma's ubiquitous direct-to-consumer TV drug ads. By saying that cholesterol levels derive from two sources—diet and family history—commercials for Lipitor and other cholesterol-reducing statins introduce the notion that genetics is probabilistic rather than deterministic in a very basic way that people can understand, he explains. “But if you say that genetic predisposition is ‘probabilistic,’ you've just lost 90% of the people.”

The limiting step in enhancing scientific literacy is not people's capacity for learning, Miller says, as much as it is interest. When Americans are diagnosed with cancer or some other life-threatening disease, “the vast number of these people go online and learn more science in the next 12 months than a typical undergraduate will ever learn. It is impressive how much people can learn with the proper motivation. We need to get people to be savvy about how to find the information and make sense of it.”

Miller urges scientists to take comfort in the fact that the majority of Americans are not anti-science, but simply don't know how exciting scientific discovery can be. “We must be cautious and not presume that our society feels strongly about what scientists do one way or another. There's a lot of work to be done for us to tell people what we do, why we do it, and why it's important,” he advises. Given the pace of biomedical discoveries in the 21st century, he adds, it's likely that more and more scientific issues will reach the public agenda. “We're going to be revisiting various versions of these questions again and again. But there's a large segment of Americans who still haven't made up their mind on these issues. We in the scientific community have to treat them seriously, talk to them, and make our arguments. This is a great opportunity for us.”

**Evolution under Attack**

As executive director of the National Center for Science Education (NCSE), Eugenie Scott leads the effort to defend the teaching of evolution in US public schools. And she's got her work cut out for her: more anti-evolution legislation was introduced in just the first six weeks of 2006—12 bills in nine states—than in any year in history. Addressing scientists and science educators at AAAS, Scott blamed the recent legislative onslaught on the legacy of an “enormously decentralized” public education system in which each of 17,000 independent school districts could teach completely different science courses. In an attempt to rectify this situation, the first National Science Education Standards were published in 1996, and many states adopted standards based on the national guidelines. Since many of the same people were involved in both efforts, Scott explained, evolution is largely included in state standards.

“What that has meant is that for the first time in many states, school districts are faced with the prospect of needing to teach evolution,” Scott said. And they must do so by the end of 2007. Ironically, this mandate comes from President George W. Bush's No Child Left Behind Act, enacted in 2002. The law effectively links annual student assessments to curriculum standards, which means that if a requirement is in the standards, it must be taught. Thanks to No Child Left Behind, the schools where Bush's fundamentalist constituents send their kids are now teaching evolution, in many cases for the first time. The result? “If you don't want evolution to be taught, you need to attack the standards,” Scott said.

And the attacks have come fast and furious, often following a similar pattern. “First, they will try to get evolution out,” she said. When that fails, they try to get “intelligent design” in. When that fails, they try to get some form of “evidence against evolution” taught, including “teach the controversy.” When anti-evolution groups say “teach the controversy,” Scott wryly pointed out, “they don't mean teach the controversy over whether birds descended from dinosaurs. They're not saying teach the controversy over sympatric and allopatric speciation. They're saying teach the controversy *as if* scientists are arguing about whether living things descended with modification from common ancestors.”

As the state education standards have come up for review over the past five years, the NCSE has increasingly encountered campaigns to teach “alternate theories.” In Dover, Pennsylvania, the school board passed a policy in 2004 that read: “Students will be made aware of gaps/problems in Darwin's theory and of other theories of evolution including, but not limited to, intelligent design. Note: Origins of life will not be taught.” Launching the first explicit legal challenge to intelligent design, parents sued the school district. At first, the legal team narrowly focused their strategy on attacking intelligent design, the team said, because they knew a judge would ask who the intelligent designer is. But since the legally problematic “gaps/problems” language was wrapped up in intelligent design in this case, the team saw an opportunity to “sink both” and head off cases down the line that used the evidence-against-evolution approach. The strategy paid off. [Fig pbio-0040167-g003]

**Figure pbio-0040167-g003:**
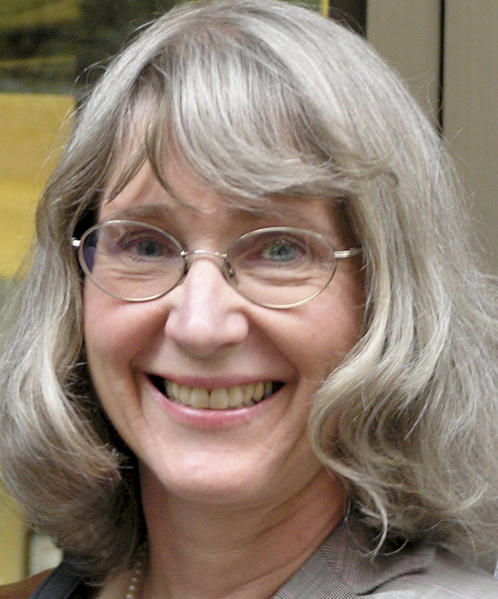
Eugenie Scott leads the battle to teach evolution in US public high schools (Photo: Steve Mirsky)

US District Judge John Jones ruled that the Dover policy violates the Establishment Clause, which mandates the separation of church and state: “In making this determination,” he wrote, “we have addressed the seminal question of whether intelligent design is science. We have concluded that it is not, and moreover that intelligent design cannot uncouple itself from its creationist, and thus religious, antecedents.”

Though buoyed by the decision, Scott has seen too many battles to let her guard down. “Intelligent design may be dead as a legal strategy but that does not mean it is dead as a popular social movement,” she said. Creationists will find other ways to get evolution out of the schools, noting that phrases like “sudden emergence theory” and “creative evolution” have already surfaced. She cautioned scientists and educators to remain vigilant in the face of ongoing challenges from the anti-evolution movement. “It's got legs,” she said. “It will evolve.”
